# Musculoskeletal Complaints in Occupational Therapists Compared to the General Population: A Cross-Sectional Study in Germany

**DOI:** 10.3390/ijerph17144916

**Published:** 2020-07-08

**Authors:** Ronny Zenker, Maria Girbig, Janice Hegewald, Irina Gilewitsch, Mandy Wagner, Albert Nienhaus, Andreas Seidler

**Affiliations:** 1 Institute and Policlinic of Occupational and Social Medicine, Faculty of Medicine Carl Gustav Carus, TU Dresden, Fetscherstr 74, 01307 Dresden, Germany; ronny.zenker@uniklinikum-dresden.de (R.Z.); janice.hegewald@tu-dresden.de (J.H.); irinagilewitsch@yahoo.de (I.G.); mandy.wagner@iqtig.org (M.W.); andreas.seidler@tu-dresden.de (A.S.); 2 Competence Center for Epidemiology and Health Services Research for Healthcare Professionals (CVcare), Institute for Health Services Research in Dermatology and Nursing (IVDP), University Medical Centre Hamburg-Eppendorf, 20246 Hamburg, Germany; a.nienhaus@uke.de; 3 Department of Occupational Medicine, Hazardous Substances and Public Health, Institution for Statutory Accident Insurance and Prevention in the Health and Welfare Services (BGW), 22089 Hamburg, Germany

**Keywords:** work-related complaints, work-related diseases, occupational health, musculoskeletal complaints, musculoskeletal diseases, occupational therapists, general population

## Abstract

Occupational therapists (OTs) are exposed to physical factors at work, which can lead to an increased risk of musculoskeletal disorders. Most studies examining musculoskeletal complaints in OT report that the neck, shoulders, and lower back are most often afflicted. The aim of the present study was to examine the impact of work as an OT (focusing on specific work tasks) on specific musculoskeletal complaints compared to the general working population. A cross-sectional study involving a self-administered standardized questionnaire was conducted from January until October 2015 in Germany. In OT and the comparison group, the highest 12-month prevalence of musculoskeletal disorders were observed for the lower back, the neck, and the shoulders. In contrast, elevated prevalence ratios (PR) were found for OT in the thumbs (PR = 2.7; 95% CI = 1.9–3.8), the wrists (PR = 1.4; 95% CI = 1.1–1.8), and the elbows (PR = 1.5; 95% CI = 1.0–2.2). OTs were particularly exposed to high exertion hand activity and several stressful postures at work. In conclusion, OTs seem to be at risk of developing work-related musculoskeletal complaints in the thumbs, wrists, and elbows. Preventive measures should help to reduce the exposures caused by highly repetitive and forceful hand activities.

## 1. Introduction

Allied health professionals are exposed to physical and psychosocial factors at work, which can lead to an increased risk of musculoskeletal disorders [1]. In Germany, the second largest occupational group within the field of allied health professionals are occupational therapists (OTs) with a workforce of about 59,000 persons [2]. Despite their importance within healthcare, little is known regarding their risks for work-related musculoskeletal complaints and diseases.

So far, there is hardly any evidence-based knowledge on work-related musculoskeletal exposures and complaints of occupational therapists in Germany. Most international studies, regarding musculoskeletal complaints in OT, find the highest prevalence in the lower back, the neck, and the shoulders [3,4,5]. Often, studies of musculoskeletal exposures compared occupational therapists to other healthcare professions (e.g., physical therapists or nurses), but an “unexposed” comparison group (unexposed to the occupational risk of occupational therapists) was not considered. Therefore, there is only little information about the particular risk of the occupational therapy professionals.

Previous studies often examined work-related risk factors and disorders of physical therapists and occupational therapists together, assuming that the specialties share a similar exposure profile [4,6,7,8]. The working conditions of both physical and occupational therapists are typically physically demanding due to patient handling tasks [1,5,8,9,10]. More specifically, both occupational groups often transfer equipment and patients [1,8,11,12], which causes stress on the spine. This stress seems to be strongly associated with lower back pain [5,10,12,13,14,15,16], as well as, neck and shoulder pain [15,17,18]. Furthermore, therapy-specific activities of both healthcare professions, such as joint mobilization and soft tissue work, are associated with increased strain on the elbow, the hands, and the wrists [13,17,19]. Additionally, repetitive tasks and an elevated workload (i.e., high frequency of treatments) might increase the risk of musculoskeletal strain injury among therapists [5,18,20].

The aim of the present study is to examine the impact of work as an occupational therapist, focusing on specific work tasks (e.g., lifting/transferring of patients, working in awkward positions), on the prevalence of specific musculoskeletal complaints compared to the general working population.

## 2. Materials and Methods

A cross-sectional study involving a self-administered questionnaire was conducted from January–October 2015 in Germany. The study was approved by the Ethics Committee of the Technical University Dresden (EK 305082014).

### 2.1. Sample Size Calculation

The calculation of the sample size was carried out using the WinEpi (Working in Epidemiologogy; free access, available under: http://winepi.unizar.es.) power analysis program [21]. With regard to existing studies, complaints of the lower back seem to be particularly relevant for the occupational therapists, so the calculations were based on an expected 12-month prevalence of lower back pain in the general population of 55% [22]. To estimate a prevalence ratio (PR) of at least 1.5 with a power of at least 80% and a significance level of 0.05 (two-sided test), we determined the random sample needed to comprise of at least 44 occupational therapists and 44 people selected from the general population. However, since detecting similar prevalence ratios for less frequent complaints requires a considerably higher sample size, we aimed to recruit approximately 300 people in each study group.

### 2.2. Participants

Employed occupational therapists (OTs) from the federal state of Saxony, Germany (age: 18 to 65 years) were asked to participate in the study. We also recruited a random selection of residents living in the state capital of Saxony (Dresden, Germany; age: 18–65 years) as the external (unexposed) comparison group (CG). Two reminders (four and eight weeks after initial contact) were sent to increase the response in all groups.

Since there is no official registration of occupational therapists in Germany, a central recruitment was not possible. To obtain a nearly representative sample of this occupational group, we contacted 150 randomly-selected outpatient occupational therapy practices in Saxony chosen from the practices listed on the website of the German Association of Occupational Therapists (DVE Deutscher Verband der Ergotherapeuten, e.V., Link: https://dve.info/service/therapeutensuche). In addition, in order to recruit OTs working in inpatient settings, we randomly selected five clinics from all the clinics in Saxony specializing in pediatrics, neurology, orthopedics, psychiatry, or psychosomatic illnesses. Five questionnaires were sent to each practice or clinic, with the information that additional questionnaires could be provided if more occupational therapists were employed at the facility. Unfortunately, the number of occupational therapists employed at each practice and clinic could not be determined, but part of our survey asked participating occupational therapists about the number of colleagues or employees working in their team. Thus, on average within our random sample about five occupational therapists worked in a practice and about 10 in a clinic. Based on this estimation approximately 750 outpatient and 50 inpatient occupational therapists were invited to participate in the study.

For the recruitment of the comparison group from the general working population, residents were randomly selected by the registration office of Dresden. One group of 300 residents was contacted and invited by mail in May 2015. An additional comparison group was recruited earlier as part of the “Physical Therapist Cohort” study using the same recruitment methods [23]. This earlier sample (*n* = 600) was invited in August 2012. In our study, the comparison group from the general working-population represents the background risk for different complaints not specific to occupational therapists. Significant differences between the groups (excess risk) are thus due to the occupational risk.

**Figure 1:**
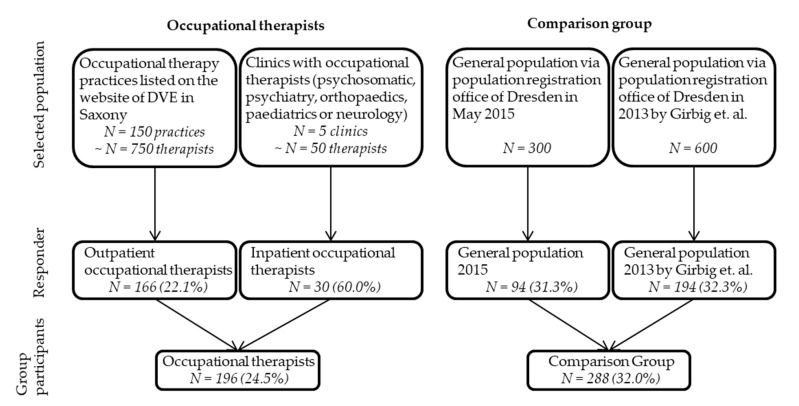
Recruitment process and response.

In total, 484 persons (196 occupational therapists and 288 control persons) completed the questionnaire. After data review and entry into SPSS no data had to be excluded. We estimated a response of 25% among occupational therapists and 32% among the comparison group. A detailed illustration of the recruitment process is shown in Figure 1.

### 2.3. Data Collection

Data collection was carried out by using a self-administered standardized questionnaire, containing a total of 131 questions with the main focus on work-related exposure and musculoskeletal complaints. The basic structure of the questionnaire was adapted from the short version of the “Physical Therapist Cohort” questionnaire [23]. Information about demographic characteristics, musculoskeletal exposures, and complaints (12-month prevalence) were collected using the following validated and established measurement tools:

- German demographic standards [24];
- Adapted EPILIFT (German Lumbar Spine Study) questions to assess musculoskeletal exposures associated with work-related postures in general, and the back [25];
- Hand activity level (HAL, [26]) ratings for exposures associated with hand activity combined (measured on scales of 0–10 where the answer possibilities were: 0 = ”Hand idle most of the time; no regular exertions” to 10 = “Rapid, steady motion/difficulty keeping up or continuous exertion”);
- Normalized peak force (nPF, [27]) for exposures associated with hand strength (measured on scales of 0–10 where 0 = ”nothing at all” and 10 = ”extremely strong”) and;
- The Nordic questionnaire (German version:) to measure musculoskeletal workload and complaints [28,29].

Combinations of HAL and nPF were considered according to the threshold limit value (TLV) [30] and action limit (AL) [31].

### 2.4. Statistical Analyses

We evaluated the received data descriptively and analytically. The descriptive analysis included the prevalence of musculoskeletal complaints related to particular body areas among occupational therapists and their work-related exposures. A Student’s *t*-test was used to test for differences in continuous variables between groups and group differences of categorical variables were tested using the Chi-square test. Prevalence ratios (PR) [32] were estimated using a binomial generalized linear regression model with a log-link function (log-binomial model) to analyze the association between work as an occupational therapist and specific complaints adjusted by age as a linear variable. All analyses were stratified by gender. Additionally, a log-binomial model with OTs categorized according to their exposure level was applied as a sensitivity analysis. We categorized the OT based on threshold limit values (TLVs) and action limits (ALs). OT with values below the AL were rated as low risk, OT with values between the AL and TLV were rated as moderate risk versus HAL and nPF above the TLV were rated as unsafe or high risk, and they were compared to the comparison group.

To test statistical significance, *p*-values (*p* < 0.05) or 95% confidence intervals were determined. All tests were two-sided and performed using IBM SPSS version 22 (IBM, Armonk, NY, USA) and SAS University Edition 2.8 9.4 M6 (SAS Institute, Cary, North Carolina, USA). Cases with missing values were dropped from the analyses by listwise deletion.

## 3. Results

### 3.1. Sociodemographic Characteristics

The sociodemographic characteristics of the study population are described in Table 1. Compared to the general working population, the group of occupational therapists consisted of more women (*p* < 0.001), younger workers (*p* < 0.001), and similar BMI (Body-Mass-Index). The occupational therapists were mainly employed in the outpatient sector.

### 3.2. Descriptive Analysis of Work-Related Exposures

**Figure 2:**
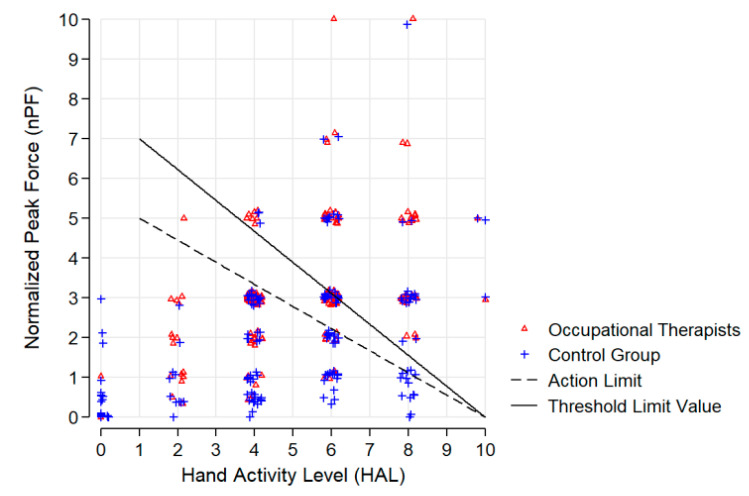
The hand activity level (HAL), normalized peak force (nPF), threshold limit value (TLV), and action limit (AL) of the study population.

Work-related exposures for musculoskeletal complaints like unfavorable postures or hand activity are shown in Supplementary Table S1. We found that occupational therapists were more frequently exposed to a higher hand activity level, higher maximum hand force, and hand activity levels exceeding threshold limit values. The mean hand activity level (HAL) for OT was 5.2 (SD = 1.8), and thus slightly above the average of the comparison group (4.8 (SD=2.6)), but the difference was not statistically significant. The mean hand force (nPF) for OT was 3.2 (SD = 1.5) and statistically significantly higher than for the comparison group with nPF=2.0 (SD = 1.8). HAL and nPF combined was over TLV for OT in 30% (*n* = 57) and between action AL and TLV for 24% (*n* = 46). This is statistically significantly higher than for CG: 20% (*n* = 33) over TLV and 10% (*n* = 16) between AL and TLV. All related values of HAL, nPF, TLV, and AL can be found in Figure 2.

Additionally OTs were more frequently exposed to stressful postures, such as bent and twisted upper body, extreme torso flexion, squatting, kneeling, heel position, or activities with hands over shoulders at work.

### 3.3. Prevalence of Musculoskeletal Complaints

Among the occupational therapists, the highest 12-month prevalence of musculoskeletal disorders pertained to the following body areas: lower back (women: 74.4%, men: 53.8%), neck/cervical spine (women: 81.0%, men: 61.5%), and the shoulders (women: 71.3%, men: 46.2%). These body areas were also reported by the comparison group to be the most frequently affected by complaints, so that no significant differences between the groups could be determined (see Table 2).

### 3.4. Prevalence Ratios of Musculoskeletal Complaints

The PRs for the 12-month prevalence of musculoskeletal complaints of all included body areas can be found in Table 3. Statistically significant increased age-adjusted PRs for female occupational therapists were found in the following body areas: the elbows (PR = 1.6 (95% CI 1.0–2.6)), the wrists (PR = 1.5 (95% CI 1.1–1.9)), and the thumbs (PR = 2.6 (95% CI 1.8–3.8)). In male occupational therapists the PR for the thumbs (PR = 3.2 (95% CI 1.4–7.2)) showed a statistically significant increase. Basically, all PRs for the upper extremities, as well as the neck/shoulders, tended to show an increased prevalence in OT.

### 3.5. Sensitivity Analysis: Prevalence Ratios with Work-Related Exposures

Age-adjusted regression models found associations between increased hand activity levels exceeding threshold limit values and the relative prevalence of musculoskeletal complaints of the upper extremity. We found no association between increased hand activity levels and the prevalence of musculoskeletal complaints of the shoulder and the neck. Occupational therapists were categorized by their threshold limit values and action limit values and compared with the complete comparison group in regression models (see Table 4).

## 4. Discussion

The main results of our study show an increased prevalence of musculoskeletal complaints in the upper extremities of occupational therapists, compared to the general working population. Particularly the relative prevalence of complaints in the thumbs (men and women), as well as the wrists and elbows (women only) were significantly increased (PR range: 1.4–3.2).

Previous research indicated that musculoskeletal complaints affecting the lower back, the neck, and the shoulders were most prevalent among occupational therapists [3,6,15,17,33,34]. We too found the highest 12-month prevalence of musculoskeletal complaints in these body areas among the occupational therapists. However, in contrast to the existing studies, our work included an adequate comparison group—members of the general working population. Within the comparison group, we also observed an increased prevalence of musculoskeletal complaints in the neck/cervical spine, the lower back, as well as, the shoulders. That is why the PRs for these regions are unremarkable. These complaints are widespread in the general population, so it is not surprising that they are also common among occupational therapists. The frequent mentioning of the lower back or lower back pain, in many previous studies with occupational therapists, probably reflects the high prevalence of lower back pain in the general population rather than a particular risk of this profession. Consequently, previous international research on the subject (without a comparison group or with an inadequate comparison group) provides very limited evidence of a work-related relationship. Future studies on this subject should, in addition to including an adequate comparison group, focus on the exposures and complaints of the upper extremities.

Another result of our study is that occupational therapists are more likely to work in unfavorable positions and with greater hand activity and hand exertion than the general working population. Such physical factors specific to work as an occupational therapist may lead to an increased risk of some forms of work-related musculoskeletal disorders like tenosynovitis, lateral epicondylitis, shoulder tendonitis, and shoulder capsulitis [35]. Our results suggest that therapy-specific manual techniques, such as joint mobilization and soft tissue work, are associated with complaints in the thumbs, the wrists, and the elbows. This is consistent with findings of previous studies [14,17,19].

Similarly, we observed hand activity levels corresponding to levels previous studies found to be associated with an increased risk for carpal tunnel syndrome [36,37]. Violante et al. observed a non-negligible risk for carpal tunnel between AL and TLV exposure limits, so these limits might not be low enough to protect some workers from complaints in the upper extremities [38]. Spielholz et al. also found the highest risk of distal upper extremity outcomes for exposures between AL and TLV (OR = 1.86, 95% CI 0.95–3.65) [30]. Within the group of the occupational therapists, we found values between AL and TLV were associated with increased risks of wrist and thumb complaints (PR = 1.7 and PR = 2.8). An increased risk of thumb complaints was found in values below AL too (PR = 2.5). This seems to support the results of Violante et al. 2016 in terms of potential impacts of low exposures, even if our results were not statistically significant.

A valid exposure measurement of the upper extremities—especially of the hands—appears to be important for future studies. In this regard, the validated HAL questionnaire [30] used in this study appears to be suitable. To objectively measure physical workloads, future studies could conceivably use, for example, the motion and force capture system CUELA (computer-assisted recording and long-term analysis of musculoskeletal load) [39]. Thus, determining work-related exposures and risk factors serves as the foundation for developing suitable preventive measures for occupational therapists and similar professional groups. Previous studies that dealt with hand or thumb complaints of therapists, recommend for example, offering ergonomic training with a focus on the correct execution of manual techniques, the use of aids and equipment, or the reduction of working hours using manual techniques [40,41].

Occupational therapists with high specific exposures, like the hand activity level and hand exertion, have significantly higher risks than those with lower exposures. This indicates an etiological relevance of these specific exposures for musculoskeletal complaints. Furthermore, these results show that occupational therapists are not a homogeneous occupational group with regard to their specific exposures. Due to the diversity of the job profile of occupational therapists, suggestions for preventive measures should focus on typical job-specific activities or activity patterns (e.g., manual therapy and joint mobilization). In this context, research on other professional groups with a similar job profile should be considered. For example, physical therapists and massage therapists also frequently use manual techniques, and to some extent have a similar exposure profile [13,42,43,44]. Likewise, information regarding break times and recovery times between patients or the number of patients per day seem to be very important, because repetitive tasks and a higher frequency of treatments could also increase the risk of musculoskeletal complaints [5,18,20].

In our study, we collected data using a questionnaire consisting of validated instruments, e.g., the Nordic questionnaire [29] to record musculoskeletal complaints. This should ensure the validity and comparability of our results to other studies. The questionnaire focused on musculoskeletal complaints in the past 12 months, so the ability of participants to remember past complaints may have caused an underreporting of older and milder complaints. Since both groups should have had similar difficulties remembering such past complaints, it is unlikely that recall bias influenced the estimated PRs. Future studies should include objective outcome measurements, such as physical (with secured medical diagnoses like ICD-10 (International Statistical Classification of Diseases and Related Health Problems) and/or radiological assessments. This could allow the investigation of reliable and differentiated diagnoses. The study presented here used core elements of the questionnaire and the same recruitment method for the comparison group as the study by Girbig et al. 2013 [23]. Including the comparison group of the Girbig et al. 2013 study increased the statistical power of the present study, and a sensitivity analysis excluding these participants had little effect on the results.

### Strength and Limitations

Within the present study we assessed occupational musculoskeletal exposures and complaints among occupational therapists including an adequate comparison group from the general working population for the first time and calculated prevalence ratios as association measures. Therefore, the present work provides a first insight at the particular risk of this healthcare profession. This will help to identify initial approaches for potential preventive measures.

We carried out the study using a cross-sectional design. Cross-sectional studies generally have the significant disadvantage that they can hardly observe variation over time, and consequently provide limited information about causal relationships. In addition, cross-sectional studies are particularly at risk of the “healthy worker effect”, because it is probable that occupational therapists with musculoskeletal disorders prematurely change professions to avoid the associated workload. This can lead to an underestimation of the PRs. This could be the reason why previous studies found increased risks of complaints among therapists with less experience [3,5,45,46]. More experienced therapists may have been more resilient and remained occupational therapists, while less-resilient therapists may have switched professions after experiencing musculoskeletal problems. Therefore, we propose the use of a longitudinal approach for future studies.

Within our study, we collected subjective data of exposures and outcomes using a questionnaire. Thus, distortions of the results are possible (e.g., reporting/response-bias, perception bias). Another limitation of this study is the low response and the associated limited internal validity. Since occupational therapists are not officially registered in Germany, the recruitment of these study participants occurred via different access paths (see Figure 1) to reflect the diversity of the occupational therapy activities. Indeed, despite considerable efforts (two reminders, incentives, etc.), the target response rate of 50% could not be achieved. Therefore, due to the low response (approximately 25% in OTs and 34% in the comparison group) selection effects are possible. It is possible that people particularly affected by the exposures and complaints assessed felt more compelled to respond to the questionnaire. Thus, an overestimation of the prevalence and the risk estimators (prevalence ratio) appears possible. Only 7% of the occupational therapists recruited were male, and while this reflects the gender distribution among occupational therapists in Germany [47], it makes it difficult to make reliable statements about the specific risks of male occupational therapists. In addition, the relevance of the study for occupational therapists working in inpatient settings is limited. The group of inpatient occupational therapists is relatively small (*n*=30) and the activities in the outpatient area are sometimes very different. Therefore, the presented results are primarily transferable to outpatient occupational therapists.

## 5. Conclusions

This study found indications of particular occupational hazards for the thumbs, elbows, and upper back resulting from typical occupational therapy activities. Previous research mostly found a high prevalence of musculoskeletal complaints in the areas of the lower back, the shoulders, and the neck of occupational therapists. In our view it is not possible to interpret these complaints as typical work-related complaints or diseases of this occupational group, because complaints in these areas of the body are widespread and highly prevalent in the general population. Future epidemiological studies should specifically consider work-related diseases of the upper extremities (especially the thumbs, the wrists, and the elbows), and if possible they should provide objective exposure and outcome measurements and use a longitudinal study design.

## Figures and Tables

**Table 1:** Sociodemographic characteristics of the study population (total: *n* = 484).

	Occupational Therapists (*n* = 196)	Comparison Group (*n* = 288)	*p* Value
Sex; *n* (%)			
Female	182 (92.9)	152 (53.3)	
Male	14 (7.1)	133 (46.7)	
Total	196	285	<0.001
Age; Mean (SD)	37.4 (9.6)	41.4 (12.8)	
Total; *n*	196	285	<0.001
BMI; Mean (SD)	25.0 (5.3)	25.2 (5.0)	
Total	194	281	0.689
Education; *n* (%)			
Secondary school	0 (0.0)	1 (0.3)	
High school	11 (5.6)	17 (5.9)	
Vocational	167 (85.2)	44 (15.3)	
University	18 (9.2)	30 (10.4)	
Missing	0 (0.0)	196 (67.4)	<0.001
Working setting (sector) ^1^; *n* (%)		not applicable	
Inpatient	23 (11.7)		
semi-residential	13 (6.6)		
outpatient	170 (86.7)		
other	31 (15.8)		

^1^ multiple answers were possible.

**Table 2:** 12-month prevalence of musculoskeletal complaints (prevalence/total (%)).

Body Area	Occupational Therapists (*n* = 196)			Comparison Group (*n* = 285)		
	Total	Women	Men	Total	Women	Men
Neck/Cervical spine	**149/187 (79.7%)**	**141/174 (81.0%)**	**8/13 (61.5%)**	**185/253 (73.1%)**	**111/141 (78.7%)**	**73/111 (65.8%)**
Shoulders	**128/184 (69.6%)**	**122/171 (71.3%)**	**6/13 (46.2%)**	**144/230 (62.6%)**	**91/127 (71.7%)**	**52/102 (51.0%)**
Elbows	41/179 (22.9%)	38/166 (22.9%)	3/13 (23.1%)	42/250 (16.8%)	23/136 (16.9%)	19/113 (16.8%)
Wrists	89/181 (49.2%)	85/168 (50.6%)	4/13 (30.8%)	69/216 (31.9%)	41/118 (34.7%)	28/98 (28.6%)
Thumbs	95/181 (52.5%)	89/168 (53.0%)	6/13 (46.2%)	36/205 (17.6%)	23/109 (21.1%)	13/96 (13.5%)
Lower back	**135/185 (73.0%)**	**128/172 (74.4%)**	**7/13 (53.8%)**	**169/249 (67.9%)**	**98/135 (72.6%)**	**70/113 (61.9%)**
Knee	66/182 (36.3%)	64/169 (37.9%)	2/13 (15.4%)	114/238 (47.9%)	57/125 (45.6%)	56/112 (50.0%)

The three highest 12-month prevalence per group are in **bold** type.

**Table 3:** Prevalence ratios of musculoskeletal complaints: occupational therapist (OT) vs. comparison group (CG; adjusted prevalence ratio (PR; 95% CI)).

Body Area	Total PR (95% CI)	Women PR (95% CI)	Men PR (95% CI)
Neck/Cervical spine	1.0 (0.9–1.1)	1.0 (0.9–1.15)	0.9 (0.6–1.4)
Shoulders	1.0 (0.9–1.2)	1.0 (0.9–1.16)	0.9 (0.5–1.7)
Elbows	1.5 (1.0–2.2)	1.6 (1.0–2.58)	1.5 (0.5–4.3)
Wrists	**1.4 (1.1–1.8)**	**1.5 (1.1–1.94)**	1.1 (0.5–2.6)
Thumbs	**2.7 (1.9–3.8)**	**2.6 (1.8–3.82)**	**3.2 (1.4–7.2)**
Lower back	1.0 (0.9–1.2)	1.0 (0.9–1.17)	0.8 (0.5–1.4)
Knee	0.8 (0.6–1.0)	0.9 (0.7–1.21)	0.3 (0.1–1.1)

Significant results are in **bold** type; adjusted for age and sex (for total).

**Table 4:** Prevalence ratios of musculoskeletal complaints (adjusted PR (95% CI)): occupational therapists grouped by exposure level.

Afflicted Body Areas	Neck PR (95% CI); *n*/*N*	Shoulders PR (95% CI); *n*/*N*	Elbows PR (95% CI); *n*/*N*	Wrists PR (95% CI); *n*/*N*	Thumbs PR (95% CI); *n*/*N*
Reference (CG)	1.0 (Ref); 185/253	1.0 (Ref); 144/230	1.0 (Ref); 42/250	1.0 (Ref); 69/216	1.0 (Ref); 36/205
below AL (OT)	1.0 (0.9–1.1) 66/86	0.9 (0.8–1.1) 53/83	1.1 (0.6–1.9); 14/82	1.2 (0.9–1.7); 35/81	**2.3 (1.5–3.5); 36/81**
between AL and TLV (OT)	1.1 (0.9–1.3) 36/42	1.0 (0.8–1.3) 31/44	1.0 (0.4–2.2); 6/41	**1.7 (1.2–2.3); 25/43**	**2.8 (1.8–4.4); 23/43**
above TLV (OT)	1.0 (0.9–1.2) 45/57	1.1 (0.9–1.3) 42/55	**2.3 (1.5–3.7); 20/54**	**1.5 (1.1–2.1); 29/55**	**3.2 (2.2–4.7); 35/55**

Significant results are in **bold** type; adjusted for age and sex; *n* = number of people with complaints; *N* = number of group study participants included in the analysis.

## Supplementary Materials

The following are available online at https://www.mdpi.com/1660-4601/17/14/4916/s1, Table S1: Work-related exposures for musculoskeletal complaints.
