# Supplementary material for: Musculoskeletal Complaints in Occupational Therapists Compared to the General Population: A Cross-Sectional Study in Germany
Source: Int J Environ Res Public Health. 2020 Jul 8;17(14):4916. doi: 10.3390/ijerph17144916 (PMC7400362; doi:10.3390/ijerph17144916)
Supplement: Supplementary file 1 [file ijerph-17-04916-s001.pdf]

Supplementary table S1. Work-related exposures for musculoskeletal complaints

	**Occupational therapists** (n=196)	**Comparison group** (n=285)	**p-value**
**Hand activity level**; n (%)			
Frequently, long pauses	19 (9.8%)	34 (20.1%)	
Steady motion, rare pauses	148 (76.3%)	102 (60.4%)	
Rapid steady motion, no pauses	27 (13.9%)	33 (19.5%)	
Total; n	194	169	0.003
**Hand force**; n (%)			
Low	43 (21.9%)	141 (62.9%)	
Moderate	107 (54.6%)	55 (24.6%)	
Big	46 (23.5%)	28 (12.5%)	
Total; n	196	224	<0.001
**Hand activity level threshold limit values**; n (%)			
Below HAL TLV	91 (46.9%)	118 (70.7%)	
Between AL and TLV	46 (23.7%)	16 (9.6%)	
Above HAL TLV	57 (29.4%)	33 (19.8%)	<0.001
**Bent and twisted**; n (%)			
Never	55 (28.2%)	51 (65.4%)	
Sometimes	119 (61.0%)	22 (28.2%)	
Often	21 (10.8%)	5 (6.4%)	
Total; n	195	78	<0.001
**Bent (75°)**; n (%)			
Never	34 (17.7%)	36 (46.8%)	
Sometimes	118 (61.5%)	28 (36.4%)	
Often	40 (20.8%)	13 (16.9%)	
Total; n	192	77	<0.001
**Bent (45°)**; n (%)			
Never	13 (6.6%)	22 (27.5%)	
Sometimes	48 (24.5%)	38 (47.5%)	
Often	135 (68.9%)	20 (25.0%)	
Total; n	196	80	<0.001
**Upright and twisted**; n (%)			
Never	18 (9.2%)	40 (51.3%)	
Sometimes	72 (36.7%)	23 (29.5%)	
Often	106 (54.1%)	15 (19.2%)	
Total; n	196	78	<0.001
**Bent (20°)**; n (%)			
Never	38 (19.4%)	18 (22.8%)	
Sometimes	90 (45.9%)	32 (40.5%)	
Often	68 (34.7%)	29 (36.7%)	
Total; n	196	79	0.685
**Extreme torso flexion (> 90°)**; n (%)			
Never	76 (39.8%)	62 (77.5%)	
Sometimes	99 (51.8%)	13 (16.3%)	
Often	16 (8.4%)	5 (6.3%)	
Total; n	191	80	<0.001
**Bent and torso flexion (45- 90°);** n (%)			
Never	15 (7.7%)	39 (48.8%)	
Sometimes	109 (56.2%)	29 (36.3%)	
Often	70 (36.1%)	12 (15.0%)	

Total; n	194	80	<0.001
**Working squatting**; n (%)			
Never	30 (15.4%)	50 (62.5%)	
Sometimes	107 (54.9%)	23 (28.7%)	
Often	58 (29.7%)	7 (8.8%)	
Total; n	195	80	<0.001
**Kneeling work**; n (%)			
Never	36 (18.5%)	57 (71.3%)	
Sometimes	109 (55.9%)	16 (20.0%)	
Often	50 (25.6%)	7 (8.8%)	
Total; n	195	80	<0.001
**Hands over shoulders**; n (%)			
Never	33 (16.9%)	47 (58.8%)	
Sometimes	121 (62.1%)	26 (32.5%)	
Often	41 (21.0%)	7 (8.8%)	
Total; n	195	80	<0.001
**Work in heel position**; n (%)			
Never	46 (23.6%)	63 (78.8%)	
Sometimes	101 (51.8%)	11 (13.8%)	
Often	48 (24.6%)	6 (7.5%)	
Total; n	195	80	<0.001
